# Opportunities for vaccine research in Europe

**DOI:** 10.1080/21645515.2015.1016680

**Published:** 2015-06-19

**Authors:** David Gancberg, Arnd Hoeveler, Alessandra Martini, Ruxandra Draghia-Akli

**Affiliations:** Directorate Health; Directorate-General for Research and Innovation; European Commission; Brussels, Belgium

**Keywords:** European union, horizon 2020, immunotherapy, vaccine

Since 1984, the European Union (EU) has been funding fundamental, applied or clinical research in all life science disciplines through its multiannual framework programmes for research and technological development (FP). The funding opportunities are fostered by different instruments (**Table 1**) and range from cooperative projects involving researchers from multiple countries, disciplines and types of organizations (academic, industry, regulators, patients' organizations, etc.) to individual research actions, infrastructure development and training activities. It also includes dedicated instruments for small and medium-size enterprises (SMEs) as well as public-public and public-private partnerships. In this paper, we focus on the support to the vaccine and immunotherapy research performed during the 7th Framework Program (FP7, 2007–2013) and discuss the current and future opportunities of the new EU funding program for research and innovation, Horizon 2020 (2014–2020).^1^

**Table 1.** FP7 and Horizon 2020 instruments for research funding

Name	Description
Research & Innovation actions	Activities aiming to establish new knowledge and/or to explore the feasibility of a new or improved technology, product, process, service or solution. For this purpose they may include basic and applied research, technology development and integration, testing and validation on a small-scale prototype in a laboratory or simulated environment. Projects may contain closely connected but limited demonstration or pilot activities aiming to show technical feasibility in a near to operational environment.
Coordination and support actions	Accompanying measures such as standardisation, dissemination, awareness-raising and communication, networking, coordination or support services, policy dialogs and mutual learning exercises and studies.
Grants of the European Research Council (ERC)^9^ to support frontier research	Starting Grant - support up-and-coming research leaders who are about to establish a proper research team and to start conducting independent research in Europe. The scheme targets promising researchers who have the proven potential of becoming independent research leaders. It will support the creation of excellent new research teams. For researchers of any nationality with 2–7 y of experience since completion of PhD (or equivalent degree) and scientific track record showing great promise. Consolidator Grant - support researchers at the stage at which they are consolidating their own independent research team or program. The scheme will strengthen independent and excellent new individual research teams that have been recently created. For researchers of any nationality with 7–12 y of experience since completion of PhD (or equivalent degree) and scientific track record showing great promise. Advanced Grant – for exceptional established research leaders of any nationality and any age to pursue ground-breaking, high-risk projects that open new directions in their respective research fields or other domains. The ERC Advanced Grant funding targets researchers who have already established themselves as independent research leaders in their own right. Proof of Concept Grant - open to researchers who have already been awarded an ERC grant. ERC grant holders can apply for this additional funding to establish the innovation potential of ideas arising from their ERC-funded frontier research projects. Synergy grant - to enable a small group of researchers and their teams to bring together complementary skills, knowledge, and resources in new ways, in order to jointly address a research problem.
Marie Skłodowska-Curie actions (MSCA)^10^	Individual Fellowships Innovative Training Networks Research and Innovation Staff Exchange Co-funding of regional, national and international programmes
SME instrument^12^	Phase 1 Feasibility study verifying the technological/practical as well as economic viability of an innovation idea with considerable novelty to the industry sector in which it is presented. The activities could, for example, comprise a business plan, risk assessment, market study, user involvement, intellectual property management, innovation strategy development, partner search, feasibility of concept and the like. Phase 2 Innovation projects that demonstrate high potential in terms of company competitiveness and growth underpinned by a strategic business plan. Activities should focus on innovation activities such as demonstration, testing, prototyping, piloting, scaling-up, miniaturisation, design, and market replication but may also include research.
Innovative Medicine Initiative (IMI)^5^	Calls for proposals aim at supporting prospective, pre-competitive pharmaceutical research and development. Stage 1: An open Call for proposals is published on the IMI website, where all interested parties from academia, SMEs, patient organisations, regulatory agencies, and large non-EFPIA companies are invited to form Applicant Consortia and to submit an Expression of Interest in response to the Call. Stage 2: Following the first stage peer review, the Applicant Consortium of the best Expression of Interest, and the EFPIA consortium already associated to the topic, are invited to form a full project consortium. IMI projects receive funding from the EU, matched by mostly in-kind contributions of EFPIA members present in the consortium. Exception: The recently published “Ebola and other filoviral hemorrhagic fevers” call for proposals is a single stage, fast track procedure. It includes 5 topics: Vaccine development phase I, II, and III; Manufacturing capability; Stability of vaccines during transport and storage; Deployment and compliance of vaccination regimens and Rapid diagnostic tests.
European and Developing Countries Clinical Trials Partnership (EDCTP)^6^	Funding of projects focusing on validation of the clinical performance and/or implementation of new or improved diagnostic tools and technologies for detection of any of the poverty-related diseases, including as co-infections. EDCTP also supports training for researchers from low- and middle-income countries who are involved in clinical research projects to develop skills for conducting clinical trials outside of an academic or public sector setting.

More than €148 million was allocated to 73 FP7 immunotherapy projects. Nineteen of those were collaborative (half of them included at least one clinical trial), amounting to €117 million. The remaining €31 million include individual grants from the European Research Council (ERC) and the Marie Sklodowska-Curie (MSC) training actions. The type of diseases targeted ranged from cancer, diabetes or allergies, to rare diseases. Typically, the projects developed new advanced technologies such as refined vectors for the production of genetically engineered cells, DNA and RNA-based vaccines, or new types of antibodies. Illustrative examples include the projects ATTACK (Adoptive engineered T-cell trials to achieve cancer killing^2^) and Cell-PID (Advanced cell-based therapies for the treatment of primary immunodeficiency^3^).

The project ADITEC (Advanced Immunization Technologies) is a significant example of a FP7 collaborative project in the vaccine field.^4^ This 5-years high impact project, receiving a €30 million EU contribution, integrates all the required expertise in immunization research (adjuvants, vectors, antigens, delivery systems, animal models, clinical trials) to study human immune responses under conditions of health and disease. ADITEC gathers 43 partners (28 academic, 13 SMEs and 2 pharmaceutical companies) from 13 different countries including the USA. After only 3 years, 9 clinical trials have been conducted or are on-going, and the project has so far delivered more than 110 peer reviewed publications (more than 70 in 2014). ADITEC is part of a large portfolio of FP7 research projects developing vaccines for infectious diseases. Overall in FP7 investments in vaccine research and development reached on average €40 million per year, with a total EU contribution of more than €317 million for the period. The majority of the funds (€234 million) was allocated to 43 collaborative projects (a third of which were performing clinical trials) dealing with antimicrobial resistance (n = 5, €25.5 million), emerging epidemics (n = 10, €41.5 million), poverty-related diseases (malaria, tuberculosis and HIV/AIDS, n = 16, €82.7 million), neglected infectious diseases (n = 9, €42.4 million) and cross-infectious diseases combining more than one of the former categories (n = 3, €42.2 million). A sector analysis of the participants revealed that higher education, research organizations and private for-profit organizations are highly represented in this field **(****Fig. 1A****)** and that the United Kingdom, France, The Netherlands and Germany are the most successful applicants in this area **(****Fig. 1B****)**. The remaining funds (€83 million) related to other specific programmes such as the ERC and the MSC grants, and the Innovative Medicines Initiative (IMI^5^). Indeed, the European Commission has explored new ways in engaging and creating original public-private (IMI) and public-public (the European and Developing Countries Clinical Trials Partnership, EDCTP^6^) partnerships in order to stimulate the development and the testing of new vaccines and medicinal products.

**Figure 1.**
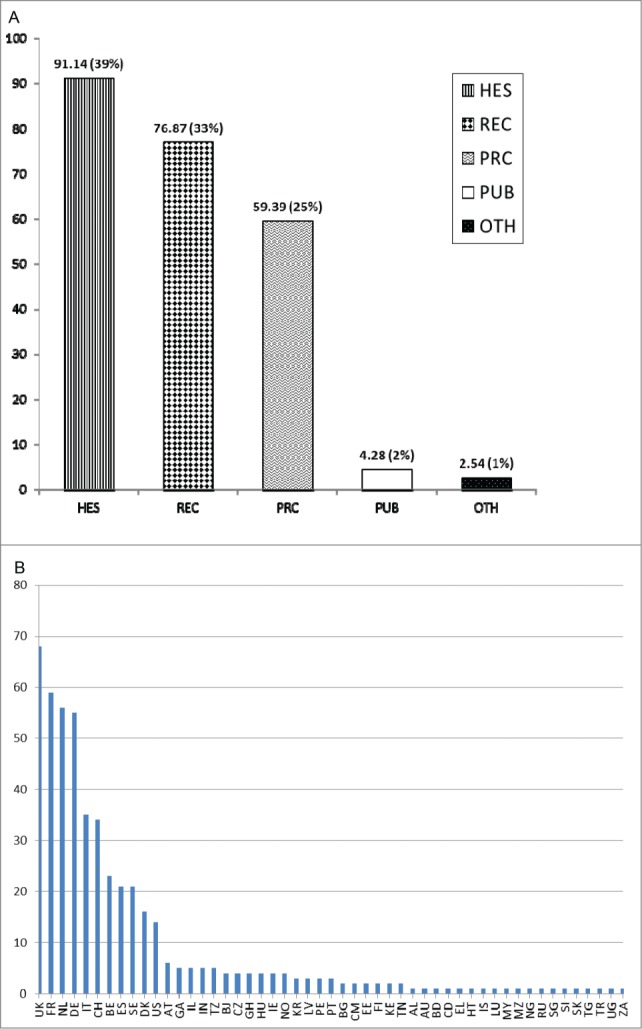
(**A**) Sum of EU contribution (in €million) per participants organization type (%) in FP7 collaborative vaccine projects for infectious diseases. Legend: HES, higher or secondary education; REC, research organization; PRC, private for profit (excluding education); PUB, public body; OTH, others. (**B**): Number of participants per country in 43 FP7 collaborative vaccine projects for infectious diseases.

Created in 2003 as a European response to the global health crisis caused by the 3 main poverty-related diseases HIV/AIDS, tuberculosis and malaria, the EDCTP aimed to accelerate the development of new or improved drugs, vaccines, microbicides and diagnostics against these 3 diseases, with a focus on clinical trials and capacity building in sub-Saharan Africa. Between 2003 and 2013, the EDCTP supported 26 research projects on vaccines, for a total of more than €64 million. With an EU contribution of €683 million for the next 10 y (2014–2023), complemented with a similar amount coming from the European Member States, for a total of almost €1.4 billion, the new phase of the program, EDCTP2, will enlarge its program to also include neglected infectious diseases, such as dengue, rabies, African trypanosomiasis or leishmaniases. All stages of clinical trials will be funded, though the main focus will remain on phase II and phase III trials.

IMI, created in 2009 and now in its second program (IMI2), is Europe's largest public-private partnership. This joint initiative between the European Commission and the European Federation of Pharmaceutical Industries and Associations (EFPIA) aims to promote the development of new, improved diagnostics and therapies for patients. IMI2, with a budget of €3.3 billion for the period 2014–2024, has just published its first calls for Horizon 2020. Vaccine research is one of the overarching priorities, as reflected by the recent launch of a €215 million call for proposals to boost research on Ebola.^7^ Half of the funds for this call will come from Horizon 2020 and the other half from the pharmaceutical companies that are members of EFPIA. Eight projects dealing with vaccine development (VSV-Ebovac, Ebovac 1 and 2), vaccine manufacture capability (Eboman), deployment of and compliance with vaccination regimens (Ebodac) and rapid diagnostic tests (Mofina, Filodiag and Ebola Modrad) were funded. 

Two previous projects on vaccines, BioVacSafe (Biomarkers for enhanced vaccine immunosafety) and Advance (Accelerated development of vaccine benefit-risk collaboration in Europe) have already been funded, for a cumulative EU contribution of over €22 million, with an additional €19 million coming from the private sector partners. While still in the preparatory phases, the next relevant projects in IMI will address zoonoses anticipation and preparedness, consistency approaches to quality control in vaccine manufacture, and pertussis vaccination.

Horizon 2020 is addressing the entire innovation cycle, from basic research to implementation in order to drive economic growth and to enhance job creation. Horizon 2020 has 3 main pillars dedicated to support Excellence in Science, Industrial Leadership and Societal Challenges. The first calls for proposals 2014–2015 in the societal challenge “Health, demographic change and wellbeing” were published in December 2013. Applicants were asked to focus on problem solving, and therefore vaccine or immunotherapy projects could find funding opportunities in various topics not specifically advertising vaccine or immunotherapy research as such. As an example, the topics PHC-16 “Tools and technologies for advanced therapies” and PHC-13 “New therapies for chronic non-communicable diseases” offered interesting opportunities. These calls are now closed but other opportunities will be published in the 2016–2017 program. The recent Ebola crisis led the EU to quickly mobilize €24.4 million from Horizon 2020 via an exceptional procedure, without a call for proposals.^8^ Among the 5 successful proposals, one includes a trial of the most advanced vaccine against Ebola developed by GlaxoSmithKline and 2 proposals that deal with the passive administration of antibodies. All the projects started on November 1st, 2014.

Notably, Horizon 2020 is open to participation of entities based in the USA due to a mutual agreement between the European Commission and the NIH.^9^ In addition to the collaborative projects of the third pillar, “societal challenges,” the first pillar of Horizon 2020 offers opportunities for frontier research science via its ERC grants,^10^ for international training networks via MSC actions^11^ and for the development of international infrastructure platforms and key enabling technologies. The second pillar will ensure support to research and innovation performers, including significant, tailored support to SMEs for which a dedicated instrument^12^ has been developed.

In conclusion, Horizon 2020 offers an even broader scope of funding opportunities for research in the field of vaccines and immunotherapy for the period 2014–2020.

## References

[1] Home - Research Participant Portal - European Commission [Internet]. Brussels (BE): funding opportunities-horizon 2020 manual. 2015- [cited 2015 June 17]. Available at: http://ec.europa.eu/research/participants/portal/desktop/en/opportunities/h2020/index.html.

[2] Attack Cancer Adoptive engineered T cell trials to achieve cancer killing [Internet]. 2013- [cited 2015 June 17]. Available at: http://www.attack-cancer.eu. 10.1089/humc.2015.2521 26086752

[3] Cell-PID Network Advanced cell-based therapies for the treatment of primary immunodeficiency. [Internet] 2011- [cited 2015 June 17]. Available at: http://www.cell-pid.eu.

[4] ADITEC Advanced immunization technologies. [Internet] 2014- [cited 2015 June 17]. Available at: http://www.aditecproject.eu.

[5] The Innovative Medicines Initiative [Internet] 2010-[cited 2015 June 17]. Available at: http://www.imi.europa.eu.

[6] EDCTP: Home The European & developing countries clinical trials partnership [Internet] 2014- [cited 2015 June 17]. Available at: http://www.edctp.org.

[7] The Innovative Medicines Initiative [Internet] 2010-[cited 2015 June 17]. Available at: http://www.imi.europa.eu/content/ebola-programme.

[8] EDCTP: Home The European & developing countries clinical trials partnership [Internet] 2014- [cited 2015 June 17]. Available at: http://www.edctp.org.

[9] Zerhouni EA, Potocnik J. European union and NIH collaborate (Letters). Science 2008; 322:1048; PMID:18974314; http://dx.doi.org/10.1126/science.1167667 18974314

[10] The European Research Council Funding and grants. [Internet] 2014- [cited 2015 June 17] Available at: http://erc.europa.eu/funding-and-grants.

[11] Horizon 2020 Marie sklodowska-curie actions. [Internet] 2014- [cited 2015 June 17] Available at: http://ec.europa.eu/programmes/horizon2020/en/h2020-section/marie-sk%C5%82odowska-curie-actions.

[12] Home - Research Participant Portal - European Commission [Internet] Brussels (BE): funding opportunities-horizon 2020 manual. 2014- [cited 2015 June 17]. HORIZON 2020 DEDICATED SME INSTRUMENT. Available at: http://ec.europa.eu/research/participants/portal/desktop/en/opportunities/h2020/calls/h2020-smeinst-2-2014.html.

